# Supplementary material for: Screen time and early adolescent mental health, academic, and social outcomes in 9- and 10- year old children: Utilizing the Adolescent Brain Cognitive Development ℠ (ABCD) Study
Source: PLoS One. 2021 Sep 8;16(9):e0256591. doi: 10.1371/journal.pone.0256591 (PMC8425530; doi:10.1371/journal.pone.0256591)
Supplement: S1 Table — Note. Significance at .05. Means and SD for weekday/weekend screen time measures given in hours. (DOCX) [file pone.0256591.s001.docx]

S1 Table. Weekday and weekend differences on screen time measures.

Weekday Weekend t statistic p-value

Mean (*SD*) Mean (*SD*)

Total Screen Time 3.46 (*3.10*) 4.62 (*3.63*) -52.31 <.001*

Parent-Report Total 2.55 (*2.59*) 3.99 (*2.66*) -61.63 <.001*

TV and Movies 1.12 (*1.10*) 1.63 (*1.28*) -50.97 <.001*

Videos 0.91 (*1.16*) 1.15 (*1.32*) -28.56 <.001*

Video Chat 0.18 (*0.49*) 0.22 (*0.61*) -10.99 <.001*

Texting 0.22 (*0.55*) 0.25 (*0.64*) -8.38 <.001*

Social Media 0.11 (*0.42*) 0.13 (*0.51*) -8.62 <.001*

Video Games 0.93 (*1.14*) 1.23 (*1.31*) -33.46 <.001*

*Note*. Significance at .05. Means and SD for weekday/weekend screen time measures given in hours.
