# Supplementary material for: Screen time and early adolescent mental health, academic, and social outcomes in 9- and 10- year old children: Utilizing the Adolescent Brain Cognitive Development ℠ (ABCD) Study
Source: PLoS One. 2021 Sep 8;16(9):e0256591. doi: 10.1371/journal.pone.0256591 (PMC8425530; doi:10.1371/journal.pone.0256591)
Supplement: S3 Table — Note. T2 Weekday = multicollinearity statistics corresponding to the interaction analyses conducted in Table 2. T3 Males = multicollinearity statistics corresponding to the regression analyses conducted in Table 3 for males only. T3 Females = multicollinearity statistics corresponding to the regression analyses conducted in Table 3 for females only. ST = screen time. R/E = race/ethnicity. SES = socioeconomic status. VIF = variance inflation factor. Tol = tolerance. Oppos. Def = oppositional defiance disorder. Cond. Dis = conduct disorder. Attn. Prob. = attention problems. Sleep Quant. = sleep quantity in hours. Sleep Qual. = sleep quality. Num. M. Fr. = number of close male friends. Num. F. Fr. = number of close female friends. (DOCX) [file pone.0256591.s003.docx]

S3 Table. Multicollinearity statistics (VIF and tolerance) for Part 1 variables.

| **T2 Weekday** | **ST VIF** | **ST Tol** | **Sex VIF** | **Sex Tol** | **R/E VIF** | **R/E Tol** | **SES VIF** | **SES Tol** |
| --- | --- | --- | --- | --- | --- | --- | --- | --- |
| **Depression** | 2.357 | .424 | 2.335 | .428 | 1.053 | .950 | 1.135 | .881 |
| **Anxiety** | 2.357 | .424 | 2.335 | .428 | 1.053 | .950 | 1.135 | .881 |
| **Internalizing** | 2.357 | .424 | 2.335 | .428 | 1.053 | .950 | 1.135 | .881 |
| **Externalizing** | 2.357 | .424 | 2.335 | .428 | 1.053 | .950 | 1.135 | .881 |
| **Oppos. Def.** | 2.357 | .424 | 2.335 | .428 | 1.053 | .950 | 1.135 | .881 |
| **Cond. Dis.** | 2.357 | .424 | 2.335 | .428 | 1.053 | .950 | 1.135 | .881 |
| **Attn. Prob.** | 2.357 | .424 | 2.335 | .428 | 1.053 | .950 | 1.135 | .881 |
| **ADHD** | 2.357 | .424 | 2.335 | .428 | 1.053 | .950 | 1.135 | .881 |
| **Acad. Perf.** | 2.338 | .428 | 2.355 | .425 | 1.056 | .947 | 1.137 | .880 |
| **Sleep Quant.** | 2.357 | .424 | 2.335 | .428 | 1.053 | .950 | 1.135 | .881 |
| **Sleep Qual.** | 2.361 | .424 | 2.335 | .428 | 1.052 | .950 | 1.135 | .881 |
| **Num. M. Fr.** | 2.360 | .424 | 2.336 | .428 | 1.053 | .950 | 1.135 | .881 |
| **Num. F. Fr.** | 2.360 | .424 | 2.336 | .428 | 1.053 | .950 | 1.135 | .881 |
| **T3 Males** | **ST VIF** | **ST Tol** | **R/E VIF** | **R/E Tol** | **SES VIF** | **SES Tol** |  |  |
| **Depression** | 1.099 | .910 | 1.048 | .954 | 1.143 | .875 |  |  |
| **Anxiety** | 1.099 | .910 | 1.048 | .954 | 1.143 | .875 |  |  |
| **Internalizing** | 1.099 | .910 | 1.048 | .954 | 1.143 | .875 |  |  |
| **Externalizing** | 1.099 | .910 | 1.048 | .954 | 1.143 | .875 |  |  |
| **Oppos. Def.** | 1.099 | .910 | 1.048 | .954 | 1.143 | .875 |  |  |
| **Cond. Dis.** | 1.099 | .910 | 1.048 | .954 | 1.143 | .875 |  |  |
| **Attn. Prob.** | 1.099 | .910 | 1.048 | .954 | 1.143 | .875 |  |  |
| **ADHD** | 1.099 | .910 | 1.048 | .954 | 1.143 | .875 |  |  |
| **Acad. Perf.** | 1.099 | .910 | 1.051 | .951 | 1.146 | .872 |  |  |
| **Sleep Quant.** | 1.099 | .910 | 1.048 | .954 | 1.143 | .875 |  |  |
| **Sleep Qual.** | 1.100 | .909 | 1.048 | .954 | 1.143 | .875 |  |  |
| **Num. M. Fr.** | 1.099 | .910 | 1.048 | .954 | 1.143 | .875 |  |  |
| **Num. F. Fr.** | 1.099 | .910 | 1.048 | .954 | 1.143 | .875 |  |  |
| **T3 Females** | **ST VIF** | **ST Tol** | **R/E VIF** | **R/E Tol** | **SES VIF** | **SES Tol** |  |  |
| **Depression** | 1.076 | .930 | 1.057 | .946 | 1.126 | .888 |  |  |
| **Anxiety** | 1.076 | .930 | 1.057 | .946 | 1.126 | .888 |  |  |
| **Internalizing** | 1.076 | .930 | 1.057 | .946 | 1.126 | .888 |  |  |
| **Externalizing** | 1.076 | .930 | 1.057 | .946 | 1.126 | .888 |  |  |
| **Oppos. Def.** | 1.076 | .930 | 1.057 | .946 | 1.126 | .888 |  |  |
| **Cond. Dis.** | 1.076 | .930 | 1.057 | .946 | 1.126 | .888 |  |  |
| **Attn. Prob.** | 1.076 | .930 | 1.057 | .946 | 1.126 | .888 |  |  |
| **ADHD** | 1.076 | .930 | 1.057 | .946 | 1.126 | .888 |  |  |
| **Acad. Perf.** | 1.075 | .930 | 1.061 | .943 | 1.127 | .887 |  |  |
| **Sleep Quant.** | 1.076 | .930 | 1.057 | .946 | 1.126 | .888 |  |  |
| **Sleep Qual.** | 1.076 | .930 | 1.057 | .946 | 1.125 | .889 |  |  |
| **Num. M. Fr.** | 1.075 | .930 | 1.058 | .946 | 1.126 | .888 |  |  |
| **Num. F. Fr.** | 1.075 | .930 | 1.057 | .946 | 1.126 | .888 |  |  |

*Note.* T2 Weekday = multicollinearity statistics corresponding to the interaction analyses conducted in Table 2. T3 Males = multicollinearity statistics corresponding to the regression analyses conducted in Table 3 for males only. T3 Females = multicollinearity statistics corresponding to the regression analyses conducted in Table 3 for females only. ST = screen time. R/E = race/ethnicity. SES = socioeconomic status. VIF = variance inflation factor. Tol = tolerance. Oppos. Def = oppositional defiance disorder. Cond. Dis = conduct disorder. Attn. Prob. = attention problems. Sleep Quant. = sleep quantity in hours. Sleep Qual. = sleep quality. Num. M. Fr. = number of close male friends. Num. F. Fr. = number of close female friends.
