# Supplementary material for: Screen time and early adolescent mental health, academic, and social outcomes in 9- and 10- year old children: Utilizing the Adolescent Brain Cognitive Development ℠ (ABCD) Study
Source: PLoS One. 2021 Sep 8;16(9):e0256591. doi: 10.1371/journal.pone.0256591 (PMC8425530; doi:10.1371/journal.pone.0256591)
Supplement: S4 Table — Note. Male participant correlations are shown across the top and in upper right triangle, female participant correlations are shown along the left side and in the lower left triangle. Grayed correlations were not significant at alpha .05. Abbreviations: Tot. = total, PR = parent report, TV = television and movies, Vid. = videos, VC = video chat, Text = texting, SM = social media, VG = video games, MG = mature games, RM = R-rated movies, Dep. = depression, Anx. = anxiety, Int. = internalizing problems, Ext. = externalizing problems, ODD = oppositional defiance disorder, CD = conduct disorder, Attn. = attention problems, AD = ADHD, AP = academic performance, ST = sleep quantity, SD = sleep quality, NCB = number of close friends who are boys, NCG = number of close friends who are girls. (DOCX) [file pone.0256591.s004.docx]

S4 Table. Correlations by sex between all variables for Part 2, weekend screen time.

|  | Tot. | PR | TV | Vid. | VC | Text | SM | VG | MG | RM | Dep. | Anx. | Int. | Ext. | ODD | CD | Attn. | AD | AP | ST | SD | NCB | NCG |
| --- | --- | --- | --- | --- | --- | --- | --- | --- | --- | --- | --- | --- | --- | --- | --- | --- | --- | --- | --- | --- | --- | --- | --- |
| Tot. |  | 0.29 | 0.64 | 0.74 | 0.37 | 0.41 | 0.31 | 0.75 | 0.35 | 0.24 | 0.08 | 0.05 | 0.07 | 0.12 | 0.10 | 0.14 | 0.14 | 0.15 | -0.16 | -0.21 | 0.12 | 0.09 | 0.09 |
| PR | 0.28 |  | 0.12 | 0.27 | 0.08 | 0.09 | 0.04 | 0.26 | 0.19 | 0.10 | 0.11 | 0.07 | 0.11 | 0.11 | 0.10 | 0.11 | 0.09 | 0.11 | -0.12 | -0.21 | 0.15 | -0.01 | 0.02 |
| TV | 0.65 | 0.14 |  | 0.26 | 0.08 | 0.12 | 0.06 | 0.29 | 0.14 | 0.14 | 0.04 | 0.04 | 0.06 | 0.09 | 0.07 | 0.09 | 0.09 | 0.10 | -0.10 | -0.10 | 0.07 | 0.03 | 0.04 |
| Vid. | 0.73 | 0.27 | 0.26 |  | 0.16 | 0.19 | 0.14 | 0.41 | 0.26 | 0.19 | 0.09 | 0.05 | 0.07 | 0.08 | 0.07 | 0.09 | 0.11 | 0.12 | -0.13 | -0.19 | 0.11 | 0.04 | 0.06 |
| VC | 0.45 | 0.11 | 0.09 | 0.21 |  | 0.25 | 0.21 | 0.17 | 0.16 | 0.08 | -0.01 | 0.00 | 0.00 | 0.04 | 0.03 | 0.07 | 0.05 | 0.05 | -0.07 | -0.07 | 0.04 | 0.07 | 0.06 |
| Text | 0.54 | 0.16 | 0.17 | 0.24 | 0.41 |  | 0.29 | 0.17 | 0.18 | 0.15 | 0.00 | 0.01 | 0.01 | 0.02 | 0.01 | 0.04 | 0.03 | 0.02 | -0.07 | -0.10 | 0.02 | 0.08 | 0.08 |
| SM | 0.42 | 0.15 | 0.09 | 0.22 | 0.24 | 0.36 |  | 0.12 | 0.18 | 0.17 | 0.02 | 0.01 | 0.01 | 0.07 | 0.08 | 0.11 | 0.06 | 0.06 | -0.08 | -0.09 | 0.05 | 0.09 | 0.11 |
| VG | 0.65 | 0.16 | 0.27 | 0.36 | 0.14 | 0.19 | 0.12 |  | 0.31 | 0.15 | 0.06 | 0.03 | 0.04 | 0.08 | 0.07 | 0.09 | 0.10 | 0.11 | -0.10 | -0.15 | 0.08 | 0.07 | 0.05 |
| MG | 0.27 | 0.16 | 0.10 | 0.18 | 0.12 | 0.17 | 0.17 | 0.23 |  | 0.39 | 0.05 | -0.01 | 0.01 | 0.10 | 0.08 | 0.13 | 0.07 | 0.08 | -0.22 | -0.21 | 0.08 | 0.08 | 0.09 |
| RM | 0.24 | 0.10 | 0.13 | 0.18 | 0.11 | 0.17 | 0.20 | 0.11 | 0.29 |  | 0.03 | -0.01 | 0.00 | 0.08 | 0.07 | 0.10 | 0.06 | 0.06 | -0.17 | -0.15 | 0.06 | 0.04 | 0.07 |
| Dep. | 0.05 | 0.09 | 0.01 | 0.06 | -0.01 | 0.02 | 0.04 | 0.05 | 0.06 | 0.04 |  | 0.57 | 0.72 | 0.48 | 0.43 | 0.41 | 0.47 | 0.38 | -0.16 | -0.15 | 0.38 | -0.05 | 0.00 |
| Anx. | 0.01 | 0.05 | -0.01 | 0.02 | -0.01 | -0.02 | 0.02 | 0.02 | 0.01 | -0.02 | 0.57 |  | 0.79 | 0.49 | 0.46 | 0.38 | 0.48 | 0.45 | -0.13 | -0.09 | 0.46 | -0.02 | 0.01 |
| Int. | 0.02 | 0.10 | 0.00 | 0.04 | -0.01 | -0.02 | 0.03 | 0.04 | 0.03 | 0.01 | 0.68 | 0.78 |  | 0.58 | 0.48 | 0.42 | 0.49 | 0.46 | -0.14 | -0.11 | 0.51 | -0.04 | 0.00 |
| Ext. | 0.13 | 0.13 | 0.08 | 0.09 | 0.05 | 0.06 | 0.09 | 0.08 | 0.09 | 0.07 | 0.48 | 0.49 | 0.59 |  | 0.83 | 0.80 | 0.59 | 0.63 | -0.25 | -0.15 | 0.46 | 0.02 | 0.04 |
| ODD | 0.10 | 0.10 | 0.07 | 0.06 | 0.03 | 0.04 | 0.08 | 0.06 | 0.06 | 0.05 | 0.44 | 0.45 | 0.49 | 0.82 |  | 0.73 | 0.57 | 0.61 | -0.20 | -0.13 | 0.42 | 0.03 | 0.03 |
| CD | 0.14 | 0.12 | 0.09 | 0.10 | 0.06 | 0.06 | 0.10 | 0.09 | 0.13 | 0.11 | 0.40 | 0.36 | 0.40 | 0.77 | 0.70 |  | 0.54 | 0.56 | -0.26 | -0.17 | 0.36 | 0.04 | 0.06 |
| Attn. | 0.13 | 0.08 | 0.07 | 0.12 | 0.04 | 0.04 | 0.06 | 0.11 | 0.09 | 0.05 | 0.43 | 0.45 | 0.47 | 0.57 | 0.54 | 0.52 |  | 0.89 | -0.38 | -0.16 | 0.43 | 0.00 | 0.05 |
| AD | 0.14 | 0.09 | 0.09 | 0.12 | 0.05 | 0.05 | 0.06 | 0.11 | 0.10 | 0.06 | 0.36 | 0.41 | 0.44 | 0.60 | 0.57 | 0.54 | 0.90 |  | -0.30 | -0.16 | 0.43 | 0.01 | 0.06 |
| AP | -0.14 | -0.13 | -0.07 | -0.13 | -0.09 | -0.08 | -0.09 | -0.05 | -0.14 | -0.12 | -0.14 | -0.08 | -0.10 | -0.22 | -0.19 | -0.24 | -0.35 | -0.29 |  | 0.22 | -0.15 | -0.02 | -0.07 |
| ST | -0.20 | -0.24 | -0.11 | -0.2 | -0.08 | -0.13 | -0.11 | -0.08 | -0.15 | -0.13 | -0.15 | -0.08 | -0.10 | -0.14 | -0.11 | -0.15 | -0.14 | -0.14 | 0.19 |  | -0.34 | 0.00 | -0.04 |
| SD | 0.10 | 0.15 | 0.06 | 0.09 | 0.01 | 0.05 | 0.05 | 0.06 | 0.06 | 0.05 | 0.37 | 0.40 | 0.49 | 0.45 | 0.39 | 0.33 | 0.44 | 0.43 | -0.14 | -0.31 |  | 0.00 | 0.05 |
| NCB | 0.07 | 0.02 | 0.03 | 0.04 | 0.05 | 0.05 | 0.07 | 0.06 | 0.10 | 0.07 | 0.00 | 0.00 | 0.02 | 0.04 | 0.03 | 0.07 | 0.06 | 0.06 | -0.04 | 0.01 | 0.03 |  | 0.45 |
| NCG | 0.05 | 0.03 | 0.02 | 0.02 | 0.07 | 0.05 | 0.05 | 0.03 | 0.02 | 0.03 | -0.03 | -0.01 | -0.02 | 0.01 | 0.01 | 0.02 | 0.02 | 0.03 | -0.03 | 0.01 | 0.01 | 0.36 |  |

Note: Male participant correlations are shown across the top and in the upper right triangle, female participant correlations are shown along the left side and in the lower left triangle. Grayed correlations were not significant at alpha .05. Abbreviations: Tot. = total screen time, PR = screen time parent report, TV = television and movies, Vid. = videos, VC = video chat, Text = texting, SM = social media, VG = video games, MG = mature games, RM = R-rated movies, Dep. = depression, Anx. = anxiety, Int. = internalizing problems, Ext. = externalizing problems, ODD = oppositional defiance disorder, CD = conduct disorder, Attn. = attention problems, AD = ADHD, AP = academic performance, ST = sleep quantity, SD = sleep quality, NCB = number of close friends who are boys, NCG = number of close friends who are girls.
