# Supplementary material for: Screen time and early adolescent mental health, academic, and social outcomes in 9- and 10- year old children: Utilizing the Adolescent Brain Cognitive Development ℠ (ABCD) Study
Source: PLoS One. 2021 Sep 8;16(9):e0256591. doi: 10.1371/journal.pone.0256591 (PMC8425530; doi:10.1371/journal.pone.0256591)
Supplement: S5 Table — Note. T4 Weekend = multicollinearity statistics corresponding to the interaction analyses conducted in Table 4. T5 Males = multicollinearity statistics corresponding to the regression analyses conducted in Table 5 for males only. T5 Females = multicollinearity statistics corresponding to the regression analyses conducted in Table 5 for females only. ST = screen time. R/E = race/ethnicity. SES = socioeconomic status. VIF = variance inflation factor. Tol = tolerance. Oppos. Def = oppositional defiance disorder. Cond. Dis = conduct disorder. Attn. Prob. = attention problems. Sleep Quant. = sleep quantity in hours. Sleep Qual. = sleep quality. Num. M. Fr. = number of close male friends. Num. F. Fr. = number of close female friends. (DOCX) [file pone.0256591.s005.docx]

S5 Table. Multicollinearity statistics (VIF and tolerance) for Part 2 variables.

| **T4 Weekend** | **ST VIF** | **ST Tol** | **Sex VIF** | **Sex Tol** | **R/E VIF** | **R/E Tol** | **SES VIF** | **SES Tol** |
| --- | --- | --- | --- | --- | --- | --- | --- | --- |
| **Depression** | 2.289 | .437 | 2.860 | .350 | 1.053 | .949 | 1.103 | .907 |
| **Anxiety** | 2.289 | .437 | 2.860 | .350 | 1.053 | .949 | 1.103 | .907 |
| **Internalizing** | 2.289 | .437 | 2.860 | .350 | 1.053 | .949 | 1.103 | .907 |
| **Externalizing** | 2.289 | .437 | 2.860 | .350 | 1.053 | .949 | 1.103 | .907 |
| **Oppos. Def.** | 2.289 | .437 | 2.860 | .350 | 1.053 | .949 | 1.103 | .907 |
| **Cond. Dis.** | 2.289 | .437 | 2.860 | .350 | 1.053 | .949 | 1.103 | .907 |
| **Attn. Prob.** | 2.289 | .437 | 2.860 | .350 | 1.053 | .949 | 1.103 | .907 |
| **ADHD** | 2.289 | .437 | 2.860 | .350 | 1.053 | .949 | 1.103 | .907 |
| **Acad. Perf.** | 2.263 | .442 | 2.867 | .349 | 1.057 | .946 | 1.106 | .905 |
| **Sleep Quant.** | 2.289 | .437 | 2.860 | .350 | 1.053 | .949 | 1.103 | .907 |
| **Sleep Qual.** | 2.291 | .436 | 2.860 | .350 | 1.053 | .950 | 1.103 | .907 |
| **Num. M. Fr.** | 2.290 | .437 | 2.860 | .350 | 1.054 | .949 | 1.103 | .907 |
| **Num. F. Fr.** | 2.289 | .437 | 2.859 | .350 | 1.053 | .949 | 1.103 | .907 |
| **T5 Males** | **ST VIF** | **ST Tol** | **R/E VIF** | **R/E Tol** | **SES VIF** | **SES Tol** |  |  |
| **Depression** | 1.061 | .942 | 1.050 | .953 | 1.105 | .905 |  |  |
| **Anxiety** | 1.061 | .942 | 1.050 | .953 | 1.105 | .905 |  |  |
| **Internalizing** | 1.061 | .942 | 1.050 | .953 | 1.105 | .905 |  |  |
| **Externalizing** | 1.061 | .942 | 1.050 | .953 | 1.105 | .905 |  |  |
| **Oppos. Def.** | 1.061 | .942 | 1.050 | .953 | 1.105 | .905 |  |  |
| **Cond. Dis.** | 1.061 | .942 | 1.050 | .953 | 1.105 | .905 |  |  |
| **Attn. Prob.** | 1.061 | .942 | 1.050 | .953 | 1.105 | .905 |  |  |
| **ADHD** | 1.061 | .942 | 1.050 | .953 | 1.105 | .905 |  |  |
| **Acad. Perf.** | 1.060 | .943 | 1.053 | .950 | 1.108 | .903 |  |  |
| **Sleep Quant.** | 1.061 | .942 | 1.050 | .953 | 1.105 | .905 |  |  |
| **Sleep Qual.** | 1.062 | .942 | 1.049 | .953 | 1.105 | .905 |  |  |
| **Num. M. Fr.** | 1.061 | .942 | 1.050 | .952 | 1.106 | .904 |  |  |
| **Num. F. Fr.** | 1.062 | .942 | 1.050 | .952 | 1.106 | .904 |  |  |
| **T5 Females** | **ST VIF** | **ST Tol** | **R/E VIF** | **R/E Tol** | **SES VIF** | **SES Tol** |  |  |
| **Depression** | 1.047 | .955 | 1.057 | .946 | 1.100 | .909 |  |  |
| **Anxiety** | 1.047 | .955 | 1.057 | .946 | 1.100 | .909 |  |  |
| **Internalizing** | 1.047 | .955 | 1.057 | .946 | 1.100 | .909 |  |  |
| **Externalizing** | 1.047 | .955 | 1.057 | .946 | 1.100 | .909 |  |  |
| **Oppos. Def.** | 1.047 | .955 | 1.057 | .946 | 1.100 | .909 |  |  |
| **Cond. Dis.** | 1.047 | .955 | 1.057 | .946 | 1.100 | .909 |  |  |
| **Attn. Prob.** | 1.047 | .955 | 1.057 | .946 | 1.100 | .909 |  |  |
| **ADHD** | 1.047 | .955 | 1.057 | .946 | 1.100 | .909 |  |  |
| **Acad. Perf.** | 1.048 | .954 | 1.060 | .943 | 1.103 | .906 |  |  |
| **Sleep Quant.** | 1.048 | .955 | 1.057 | .946 | 1.100 | .909 |  |  |
| **Sleep Qual.** | 1.047 | .955 | 1.057 | .946 | 1.100 | .909 |  |  |
| **Num. M. Fr.** | 1.047 | .955 | 1.057 | .946 | 1.100 | .909 |  |  |
| **Num. F. Fr.** | 1.047 | .955 | 1.057 | .946 | 1.100 | .909 |  |  |

*Note.* T4 Weekend = multicollinearity statistics corresponding to the interaction analyses conducted in Table 4. T5 Males = multicollinearity statistics corresponding to the regression analyses conducted in Table 5 for males only. T5 Females = multicollinearity statistics corresponding to the regression analyses conducted in Table 5 for females only. ST = screen time. R/E = race/ethnicity. SES = socioeconomic status. VIF = variance inflation factor. Tol = tolerance. Oppos. Def = oppositional defiance disorder. Cond. Dis = conduct disorder. Attn. Prob. = attention problems. Sleep Quant. = sleep quantity in hours. Sleep Qual. = sleep quality. Num. M. Fr. = number of close male friends. Num. F. Fr. = number of close female friends.
