# Supplementary material for: Screen time and early adolescent mental health, academic, and social outcomes in 9- and 10- year old children: Utilizing the Adolescent Brain Cognitive Development ℠ (ABCD) Study
Source: PLoS One. 2021 Sep 8;16(9):e0256591. doi: 10.1371/journal.pone.0256591 (PMC8425530; doi:10.1371/journal.pone.0256591)
Supplement: S6 Table — Note. Starred regressions are significant at alpha .05. (DOCX) [file pone.0256591.s006.docx]

S6 Table. Depression regressed on various types of weekday screen time for Part 1, controlling for SES and race/ethnicity, separated by sex.

Standardized Partial

Beta t statistic p-value Std. Err. Correlation

Males (*N*=6111)

Parent Report 0.036 2.67 .008* .038 .036

TV and Movies 0.035 2.58 .010* .077 .035

Videos 0.046 3.40 .001* .072 .046

Video Chat -0.020 -1.51 .131 .198 -.020

Texting -0.012 -0.91 .361 .183 -.012

Social Media 0.003 0.24 .810 .248 .003

Video Games 0.018 1.36 .175 .069 .018

Mature Video Games 0.005 0.33 .742 .090 .004

R-rated Movies 0.000 0.01 .992 .130 .000

Females (*N*=5613)

Parent Report 0.049 3.44 .001* .034 .048

TV and Movies 0.015 1.10 .272 .065 .015

Videos 0.031 2.20 .028* .064 .031

Video Chat -0.008 -0.57 .572 .154 -.008

Texting -0.001 -0.09 .930 .128 -.001

Social Media 0.009 0.62 .539 .180 .009

Video Games 0.031 2.24 .025* .077 .031

Mature Video Games 0.021 1.45 .146 .119 .020

R-related Movies 0.008 0.57 .572 .121 .008

*Note*. Starred regressions are significant at alpha .05.
