# Supplementary material for: Screen time and early adolescent mental health, academic, and social outcomes in 9- and 10- year old children: Utilizing the Adolescent Brain Cognitive Development ℠ (ABCD) Study
Source: PLoS One. 2021 Sep 8;16(9):e0256591. doi: 10.1371/journal.pone.0256591 (PMC8425530; doi:10.1371/journal.pone.0256591)
Supplement: S7 Table — Note. Starred regressions are significant at alpha .05. (DOCX) [file pone.0256591.s007.docx]

S7 Table. Anxiety regressed on various types of weekday screen time for Part 1, controlling for SES and race/ethnicity, separated by sex.

Standardized Partial

Beta t statistic p-value Std. Err. Correlation

Males (*N*=6111)

Parent Report 0.019 1.35 .178 .037 .018

TV and Movies 0.013 0.95 .342 .077 .013

Videos 0.034 2.45 .012* .071 .033

Video Chat 0.000 0.01 .991 .196 .000

Texting 0.007 0.51 .607 .182 .007

Social Media 0.004 0.30 .767 .246 .004

Video Games 0.016 1.15 .249 .069 .015

Mature Video Games -0.010 0.71 .477 .089 -.010

R-rated Movies -0.008 -0.56 .573 .129 -.008

Females (*N*=5613)

Parent Report 0.021 1.44 .150 .040 .020

TV and Movies -0.009 -0.65 .514 .075 -.009

Videos 0.015 1.04 .298 .075 .015

Video Chat -0.019 -1.35 .179 .180 -.019

Texting -0.016 -1.13 .257 .150 -.016

Social Media 0.001 0.05 .958 .210 .001

Video Games 0.012 0.86 .389 .090 .012

Mature Video Games 0.000 -0.03 .977 .139 .000

R-rated Movies -0.023 -1.61 .107 .142 -.023

*Note*. Starred regressions are significant at alpha .05.
