# Supplementary material for: Screen time and early adolescent mental health, academic, and social outcomes in 9- and 10- year old children: Utilizing the Adolescent Brain Cognitive Development ℠ (ABCD) Study
Source: PLoS One. 2021 Sep 8;16(9):e0256591. doi: 10.1371/journal.pone.0256591 (PMC8425530; doi:10.1371/journal.pone.0256591)
Supplement: S8 Table — Note. Starred regressions are significant at alpha .05. (DOCX) [file pone.0256591.s008.docx]

S8 Table. Internalizing symptoms regressed on various types of weekday screen time for Part 1, controlling for SES and race/ethnicity, separated by sex.

Standardized Partial

Beta t statistic p-value Std. Err. Correlation

Males (*N*=6111)

Parent Report 0.033 2.38 .017* .064 .032

TV and Movies 0.038 2.81 .005* .132 .038

Videos 0.053 3.89 <.001* .123 .052

Video Chat -0.008 -0.57 .568 .339 -.008

Texting 0.003 0.22 .827 .313 .003

Social Media 0.001 0.05 .958 .425 .001

Video Games 0.016 1.16 .246 .118 .016

Mature Video Games 0.001 0.07 .948 .154 .001

R-rated Movies -0.008 -0.58 .565 .222 -.008

Females (*N*=5613)

Parent Report 0.054 3.79 <.001* .073 .053

TV and Movies 0.006 0.46 .647 .137 .006

Videos 0.021 1.47 .142 .136 .020

Video Chat -0.024 -1.74 .081 .326 -.024

Texting -0.029 -2.05 .041* .271 -.029

Social Media -0.004 -0.29 .776 .380 -.004

Video Games 0.022 1.58 .114 .164 .022

Mature Video Games 0.007 0.52 .604 .252 .007

R-rated Movies -0.008 -0.57 .566 .257 -.008

*Note*. Starred regressions are significant at alpha .05.
