# Supplementary material for: Screen time and early adolescent mental health, academic, and social outcomes in 9- and 10- year old children: Utilizing the Adolescent Brain Cognitive Development ℠ (ABCD) Study
Source: PLoS One. 2021 Sep 8;16(9):e0256591. doi: 10.1371/journal.pone.0256591 (PMC8425530; doi:10.1371/journal.pone.0256591)
Supplement: S9 Table — Note. Starred regressions are significant at alpha .05. (DOCX) [file pone.0256591.s009.docx]

S9 Table. Externalizing symptoms regressed on various types of weekday screen time for Part 1, controlling for SES and race/ethnicity, separated by sex.

Standardized Partial

Beta t statistic p-value Std. Err. Correlation

Males (*N*=6111)

Parent Report 0.023 1.73 .083 .064 .023

TV and Movies 0.061 4.54 <.001* .130 .061

Videos 0.055 4.07 <.001* .121 .054

Video Chat 0.020 1.50 .135 .334 .020

Texting 0.012 0.91 .365 .309 .012

Social Media 0.046 3.48 <.001* .418 .047

Video Games 0.043 3.19 .001* .117 .043

Mature Video Games 0.068 4.91 <.001* .152 .066

R-rated Movies 0.053 3.93 <.001* .219 .053

Females (*N*=5613)

Parent Report 0.075 5.31 <.001* .068 .073

TV and Movies 0.058 4.13 <.001* .127 .057

Videos 0.073 5.15 <.001* .127 .072

Video Chat 0.035 2.54 .011* .303 .035

Texting 0.028 2.04 .042* .253 .028

Social Media 0.062 4.44 <.001* .354 .062

Video Games 0.050 3.59 <.001* .152 .050

Mature Video Games 0.052 3.72 <.001* .235 .052

R-rated Movies 0.051 3.64 <.001* .239 .051

*Note*. Starred regressions are significant at alpha .05.
