# Supplementary material for: Screen time and early adolescent mental health, academic, and social outcomes in 9- and 10- year old children: Utilizing the Adolescent Brain Cognitive Development ℠ (ABCD) Study
Source: PLoS One. 2021 Sep 8;16(9):e0256591. doi: 10.1371/journal.pone.0256591 (PMC8425530; doi:10.1371/journal.pone.0256591)
Supplement: S10 Table — Note. Starred regressions are significant at alpha .05. (DOCX) [file pone.0256591.s010.docx]

S10 Table. Oppositional defiance disorder regressed on various types of weekday screen time for Part 1, controlling for SES and race/ethnicity, separated by sex.

Standardized Partial

Beta t statistic p-value Std. Err. Correlation

Males (*N*=6111)

Parent Report 0.018 1.31 .192 .035 .018

TV and Movies 0.043 3.19 .001*  .072 .043

Videos 0.050 3.69 <.001* .067 .049

Video Chat 0.025 1.89 .059 .184 .025

Texting 0.015 1.11 .266 .170 .015

Social Media 0.061 4.57 <.001* .231 .061

Video Games 0.038 2.77 .006* .064 .037

Mature Video Games 0.055 3.94 <.001* .084 .053

R-rated Movies 0.058 4.25 <.001* .121 .057

Females (*N*=5613)

Parent Report 0.047 3.30 .001* .034 .046

TV and Movies 0.051 3.64 <.001* .063 .051

Videos 0.063 4.41 <.001* .063 .061

Video Chat 0.026 1.85 .065 .151 .026

Texting 0.038 2.74 .006* .126 .038

Social Media 0.058 4.12 <.001* .176 .057

Video Games 0.036 2.60 .009* .076 .036

Mature Video Games 0.036 2.53 .012* .117 .035

R-rated Movies 0.043 3.06 .002* .119 .043

*Note*. Starred regressions are significant at alpha .05.
