# Supplementary material for: Screen time and early adolescent mental health, academic, and social outcomes in 9- and 10- year old children: Utilizing the Adolescent Brain Cognitive Development ℠ (ABCD) Study
Source: PLoS One. 2021 Sep 8;16(9):e0256591. doi: 10.1371/journal.pone.0256591 (PMC8425530; doi:10.1371/journal.pone.0256591)
Supplement: S11 Table — Note. Starred regressions are significant at alpha .05. (DOCX) [file pone.0256591.s011.docx]

S11 Table. Conduct disorder regressed on various types of weekday screen time for Part 1, controlling for SES and race/ethnicity, separated by sex.

Standardized Partial

Beta t statistic p-value Std. Err. Correlation

Males (*N*=6111)

Parent Report 0.029 2.19 .029* .033 .029

TV and Movies 0.068 5.14 <.001* .069 .069

Videos 0.060 4.48 <.001* .064 .060

Video Chat 0.029 2.19 .029* .176 .029

Texting 0.026 1.96 .050 .162 .026

Social Media 0.072 5.43 <.001* .220 .073

Video Games 0.057 4.23 <.001* .061 .057

Mature Video Games 0.076 5.55 <.001* .080 .074

R-rated Movies 0.071 5.30 <.001* .115 .071

Females (*N*=5613)

Parent Report 0.065 4.62 <.001* .036 .065

TV and Movies 0.079 5.67 <.001* .067 .079

Videos 0.074 5.25 <.001* .067 .073

Video Chat 0.051 3.71 <.001* .161 .052

Texting 0.029 2.07 .030* .134 .029

Social Media 0.066 4.75 <.001* .188 .066

Video Games 0.060 4.35 <.001* .081 .061

Mature Video Games 0.075 5.37 <.001* .124 .075

R-rated Movies 0.082 5.89 <.001* .127 .082

*Note*. Starred regressions are significant at alpha .05.
