# Supplementary material for: Screen time and early adolescent mental health, academic, and social outcomes in 9- and 10- year old children: Utilizing the Adolescent Brain Cognitive Development ℠ (ABCD) Study
Source: PLoS One. 2021 Sep 8;16(9):e0256591. doi: 10.1371/journal.pone.0256591 (PMC8425530; doi:10.1371/journal.pone.0256591)
Supplement: S12 Table — Note. Starred regressions are significant at alpha .05. (DOCX) [file pone.0256591.s012.docx]

S12 Table. Attention problems regressed on various types of weekday screen time for Part 1, controlling for SES and race/ethnicity, separated by sex.

Standardized Partial

Beta t statistic p-value Std. Err. Correlation

Males (*N*=6111)

Parent Report 0.021 1.54 .123 .039 .021

TV and Movies 0.059 4.38 <.001* .080 .059

Videos 0.064 4.69 <.001* .074 .063

Video Chat 0.019 1.41 .158 .204 .019

Texting 0.005 0.39 .697 .188 .005

Social Media 0.032 2.39 .017* .255 .032

Video Games 0.051 3.76 <.001* .071 .050

Mature Video Games 0.033 2.36 .018* .093 .032

R-rated Movies 0.040 2.92 .003* .134 .039

Females (*N*=5613)

Parent Report 0.032 2.25 .024* .040 .032

TV and Movies 0.039 2.76 .006* .074 .038

Videos 0.077 5.41 <.001* .074 .075

Video Chat 0.017 1.24 .215 .178 .017

Texting 0.029 2.06 .040* .148 .029

Social Media 0.022 1.56 .120 .207 .022

Video Games 0.072 5.17 <.001* .089 .072

Mature Video Games 0.056 3.93 <.001* .137 .055

R-rated Movies 0.030 2.14 .032* .140 .030

*Note*. Starred regressions are significant at alpha .05.
