# Supplementary material for: Screen time and early adolescent mental health, academic, and social outcomes in 9- and 10- year old children: Utilizing the Adolescent Brain Cognitive Development ℠ (ABCD) Study
Source: PLoS One. 2021 Sep 8;16(9):e0256591. doi: 10.1371/journal.pone.0256591 (PMC8425530; doi:10.1371/journal.pone.0256591)
Supplement: S13 Table — Note. Starred regressions are significant at alpha .05. (DOCX) [file pone.0256591.s013.docx]

S13 Table. ADHD regressed on various types of weekday screen time for Part 1, controlling for SES and race/ethnicity, separated by sex.

Standardized Partial

Beta t statistic p-value Std. Err. Correlation

Males (*N*=6111)

Parent Report 0.027 2.00 .045* .036 .027

TV and Movies 0.078 5.75 <.001* .074 .077

Videos 0.081 5.94 <.001* .069 .079

Video Chat 0.024 1.79 .074 .189 .024

Texting 0.005 0.36 .718 .175 .005

Social Media 0.042 3.13 .002* .238 .042

Video Games 0.066 4.86 <.001* .066 .065

Mature Video Games 0.045 3.23 .001* .086 .043

R-rated Movies 0.043 3.18 .001* .124 .043

Females (*N*=5613)

Parent Report 0.042 2.94 .003* .036 .041

TV and Movies 0.050 3.57 <.001* .067 .050

Videos 0.087 6.12 <.001* .066 .085

Video Chat 0.025 1.78 .076 .159 .025

Texting 0.042 2.98 .003* .133 .042

Social Media 0.025 1.83 .068 .186 .025

Video Games 0.075 5.39 <.001* .080 .075

Mature Video Games 0.072 5.11 <.001* .123 .071

R-rated Movies 0.037 2.61 .009* .126 .036

*Note*. Starred regressions are significant at alpha .05.
