# Supplementary material for: Screen time and early adolescent mental health, academic, and social outcomes in 9- and 10- year old children: Utilizing the Adolescent Brain Cognitive Development ℠ (ABCD) Study
Source: PLoS One. 2021 Sep 8;16(9):e0256591. doi: 10.1371/journal.pone.0256591 (PMC8425530; doi:10.1371/journal.pone.0256591)
Supplement: S14 Table — Note. Starred regressions are significant at alpha .05. (DOCX) [file pone.0256591.s014.docx]

S14 Table. Grades regressed on various types of weekday screen time for Part 1, controlling for SES and race/ethnicity, separated by sex.

Standardized Partial

Beta t statistic p-value Std. Err. Correlation

Males (*N*=6111)

Parent Report -0.078 -5.73 <.001* .005 -.080

TV and Movies -0.050 -3.69 <.001*  .010 -.051

Videos -0.068 -5.02 <.001* .009 -.070

Video Chat -0.030 -2.28 .023* .026 -.032

Texting -0.051 -3.82 <.001* .024 -.053

Social Media -0.045 -3.37 .001* .032 -.047

Video Games -0.093 -6.87 <.001* .009 -.096

Mature Video Games -0.142 -10.32 <.001* .012 -.143

R-rated Movies -0.104 -7.66 <.001* .017 -.107

Females (*N*=5613)

Parent Report -0.091 -6.41 <.001* .005 -.093

TV and Movies -0.085 -6.05 <.001* .010 -.087

Videos -0.086 -6.05 <.001* .010 -.087

Video Chat -0.041 -2.93 .003* .023 -.042

Texting -0.064 -4.57 <.001* .019 -.066

Social Media -0.054 -3.87 <.001* .026 -.056

Video Games -0.062 -4.41 <.001* .011 -.064

Mature Video Games -0.073 -5.20 <.001* 0.17 -.075

R-rated Movies -0.065 -4.61 <.001* .018 -.067

*Note*. Starred regressions are significant at alpha .05.
