# Supplementary material for: Screen time and early adolescent mental health, academic, and social outcomes in 9- and 10- year old children: Utilizing the Adolescent Brain Cognitive Development ℠ (ABCD) Study
Source: PLoS One. 2021 Sep 8;16(9):e0256591. doi: 10.1371/journal.pone.0256591 (PMC8425530; doi:10.1371/journal.pone.0256591)
Supplement: S15 Table — Note. Starred regressions are significant at alpha .05. (DOCX) [file pone.0256591.s015.docx]

S15 Table. Average nightly hours of sleep regressed on various types of weekday screen time for Part 1, controlling for SES and race/ethnicity, separated by sex.

Standardized Partial

Beta t statistic p-value Std. Err. Correlation

Males (*N*=6111)

Parent Report -0.147 -11.40 <.001* .005 -.152

TV and Movies -0.088 -6.77 <.001* .010 -.090

Videos -0.138 -10.68 <.001* .009 -.142

Video Chat -0.053 -4.16 <.001* .025 -.056

Texting -0.070 -5.48 <.001* .023 -.073

Social Media -0.056 -4.34 <.001* .031 -.058

Video Games -0.123 -9.49 <.001* .009 -.126

Mature Video Games -0.132 -9.98 <.001* .011 -.133

R-rated Movies -0.099 -7.60 <.001* .016 -.101

Females (*N*=5613)

Parent Report -0.165 -12.34 <.001* .005 -.170

TV and Movies -0.074 -5.52 <.001* .010 -.077

Videos -0.146 -10.83 <.001* .010 -.149

Video Chat -0.037 -2.82 .005* .023 -.039

Texting -0.076 -5.70 <.001* .020 -.079

Social Media -0.102 -7.71 <.001* .027 -.107

Video Games -0.053 -3.99 <.001* .012 -.056

Mature Video Games -0.094 -6.95 <.001* .018 -.096

R-rated Movies -0.075 -5.60 <.001* .018 -.078

*Note*. Starred regressions are significant at alpha .05.
