# Supplementary material for: Screen time and early adolescent mental health, academic, and social outcomes in 9- and 10- year old children: Utilizing the Adolescent Brain Cognitive Development ℠ (ABCD) Study
Source: PLoS One. 2021 Sep 8;16(9):e0256591. doi: 10.1371/journal.pone.0256591 (PMC8425530; doi:10.1371/journal.pone.0256591)
Supplement: S16 Table — Note. Starred regressions are significant at alpha .05. (DOCX) [file pone.0256591.s016.docx]

S16 Table. Sleep disorder score regressed on various types of weekday screen time for Part 1, controlling for SES and race/ethnicity, separated by sex.

Standardized Partial

Beta t statistic p-value Std. Err. Correlation

Males (*N*=6111)

Parent Report 0.062 4.53 <.001* .050 .061

TV and Movies 0.045 3.34 .001* .103 .045

Videos 0.074 5.47 <.001* .095 .073

Video Chat 0.025 1.86 .063 .262 .025

Texting 0.019 1.39 .166 .243 .019

Social Media 0.032 2.39 .017* .329 .032

Video Games 0.050 3.68 <.001* .092 .049

Mature Video Games 0.054 3.84 <.001* .120 .051

R-rated Movies 0.036 2.63 .009* .172 .035

Females (*N*=5613)

Parent Report 0.097 6.86 <.001* .054 .096

TV and Movies 0.036 2.54 .011* .102 .035

Videos 0.063 4.38 <.001* .102 .061

Video Chat -0.004 -0.28 .781 .244 -.004

Texting 0.016 1.17 .240 .203 .016

Social Media 0.034 2.46 .014* .285 .034

Video Games 0.037 2.61 .009* .123 .036

Mature Video Games 0.034 2.42 .015* .189 .034

R-rated Movies 0.028 1.96 .050 .192 .027

*Note*. Starred regressions are significant at alpha .05.
