# Supplementary material for: Screen time and early adolescent mental health, academic, and social outcomes in 9- and 10- year old children: Utilizing the Adolescent Brain Cognitive Development ℠ (ABCD) Study
Source: PLoS One. 2021 Sep 8;16(9):e0256591. doi: 10.1371/journal.pone.0256591 (PMC8425530; doi:10.1371/journal.pone.0256591)
Supplement: S17 Table — Note. Starred regressions are significant at alpha .05. (DOCX) [file pone.0256591.s017.docx]

S17 Table. Number of close friends who are boys regressed on various types of weekday screen time for Part 1, controlling for SES and race/ethnicity, separated by sex.

Standardized Partial

Beta t statistic p-value Std. Err. Correlation

Males (*N*=6111)

Parent Report -0.010 -0.71 .480 .040 -.010

TV and Movies 0.027 2.00 .046* .083 .027

Videos 0.052 3.80 <.001* .077 .051

Video Chat 0.074 5.53 <.001* .212 .074

Texting 0.078 5.75 <.001* .196 .077

Social Media 0.092 6.81 <.001* .265 .091

Video Games 0.057 4.14 <.001* .074 .055

Mature Video Games 0.081 5.74 <.001* .096 .077

R-rated Movies 0.029 2.11 .035* .139 .028

Females (*N*=5613)

Parent Report -0.013 -0.93 .355 .018 -.013

TV and Movies 0.007 0.51 .608 .033 .007

Videos 0.034 2.38 .017* .033 .033

Video Chat 0.057 4.09 <.001* .078 .057

Texting 0.031 2.24 .025* .065 .031

Social Media 0.038 2.69 .007* .092 .037

Video Games 0.042 2.99 .003* .039 .042

Mature Video Games 0.101 7.15 <.001* .060 .099

R-rated Movies 0.075 5.27 <.001* .061 .073

*Note*. Starred regressions are significant at alpha .05.
