# Supplementary material for: Screen time and early adolescent mental health, academic, and social outcomes in 9- and 10- year old children: Utilizing the Adolescent Brain Cognitive Development ℠ (ABCD) Study
Source: PLoS One. 2021 Sep 8;16(9):e0256591. doi: 10.1371/journal.pone.0256591 (PMC8425530; doi:10.1371/journal.pone.0256591)
Supplement: S18 Table — Note. Starred regressions are significant at alpha .05. (DOCX) [file pone.0256591.s018.docx]

S18 Table. Number of close friends who are girls regressed on various types of weekday screen time for Part 1, controlling for SES and race/ethnicity, separated by sex.

Standardized Partial

Beta t statistic p-value Std. Err. Correlation

Males (*N*=6111)

Parent Report -0.001 -0.08 .939 .028 -.001

TV and Movies 0.006 0.45 .655 .057 .006

Videos 0.040 2.95 .003 .053 .040

Video Chat 0.099 7.37 <.001* .146 .098

Texting 0.087 6.51 <.001* .135 .087

Social Media 0.147 11.01 <.001* .182 .146

Video Games 0.030 2.17 .030* .051 .029

Mature Video Games 0.051 3.62 <.001* .067 .048

R-rated Movies 0.040 2.93 .003* .096 .039

Females (*N*=5613)

Parent Report 0.009 0.60 .549 .051 .008

TV and Movies 0.030 2.15 .031* .096 .030

Videos 0.027 1.88 .060 .096 .026

Video Chat 0.105 7.57 <.001* .228 .105

Texting 0.059 4.23 <.001* .191 .059

Social Media 0.017 1.23 .221 .269 .017

Video Games 0.026 1.87 .062 .115 .026

Mature Video Games 0.037 2.62 .009* .177 .036

R-rated Movies 0.038 2.66 .008* .181 .037

*Note*. Starred regressions are significant at alpha .05.
