# Supplementary material for: Screen time and early adolescent mental health, academic, and social outcomes in 9- and 10- year old children: Utilizing the Adolescent Brain Cognitive Development ℠ (ABCD) Study
Source: PLoS One. 2021 Sep 8;16(9):e0256591. doi: 10.1371/journal.pone.0256591 (PMC8425530; doi:10.1371/journal.pone.0256591)
Supplement: S19 Table — Note. Starred regressions are significant at alpha .05. (DOCX) [file pone.0256591.s019.docx]

S19 Table. Depression regressed on various types of weekend screen time for Part 2, controlling for SES and race/ethnicity, separated by sex.

Standardized Partial

Beta t statistic p-value Std. Err. Correlation

Males (*N*=6071)

Parent Report 0.082 6.12 <.001* .034 .082

TV and Movies 0.025 1.89 .059 .066 .025

Videos 0.054 4.01 <.001* .063 .054

Video Chat -0.027 -2.06 .040* .177 -.028

Texting -0.015 -1.15 .251 .176 -.015

Social Media 0.006 0.42 .675 .246 .006

Video Games 0.040 2.98 .003* .061 .040

Mature Video Games 0.002 0.15 .883 .091 .002

R-rated Movies -0.008 -0.62 .534 .131 -.008

Females (*N*=5598)

Parent Report 0.086 6.10 <.001* .030 .085

TV and Movies -0.005 -0.33 .745 .055 -.005

Videos 0.031 2.18 .030* .056 .030

Video Chat -0.017 -1.25 .211 .132 -.017

Texting 0.003 0.25 .803 .119 .003

Social Media 0.027 1.95 .051 .144 .027

Video Games 0.028 2.04 .042* .067 .028

Mature Video Games 0.023 1.64 .101 .119 .023

R-rated Movies 0.005 0.35 .724 .121 .005

*Note*. Starred regressions are significant at alpha .05.
