# Supplementary material for: Screen time and early adolescent mental health, academic, and social outcomes in 9- and 10- year old children: Utilizing the Adolescent Brain Cognitive Development ℠ (ABCD) Study
Source: PLoS One. 2021 Sep 8;16(9):e0256591. doi: 10.1371/journal.pone.0256591 (PMC8425530; doi:10.1371/journal.pone.0256591)
Supplement: S20 Table — Note. Starred regressions are significant at alpha .05. (DOCX) [file pone.0256591.s020.docx]

S20 Table. Anxiety regressed on various types of weekend screen time for Part 2, controlling for SES and race/ethnicity, separated by sex.

Standardized Partial

Beta t statistic p-value Std. Err. Correlation

Males (*N*=6071)

Parent Report 0.063 4.62 <.001*  .034 .062

TV and Movies 0.041 3.04 .002* .066 .041

Videos 0.046 3.39 .001* .063 .046

Video Chat 0.001 0.05 .958 .175 .001

Texting 0.013 0.95 .342 .175 .013

Social Media 0.010 0.75 .456 .244 .010

Video Games 0.030 2.24 .025* .061 .030

Mature Video Games -0.015 -1.09 .278 .090 -.015

R-rated Movies -0.012 -0.90 .368 .130 -.012

Females (*N*=5598)

Parent Report 0.054 3.81 <.001* .036 .053

TV and Movies -0.018 -1.31 .190 .065 -.018

Videos 0.012 0.87 .385 .065 .012

Video Chat -0.010 -0.71 .481 .154 -.010

Texting -0.019 -1.33 .185 .139 -.019

Social Media 0.015 1.08 .281 .168 .015

Video Games 0.018 1.27 .203 .078 .018

Mature Video Games -0.002 -0.15 .877 .139 -.002

R-rated Movies -0.023 -1.59 .113 .141 -.022

*Note*. Starred regressions are significant at alpha .05.
