# Supplementary material for: Screen time and early adolescent mental health, academic, and social outcomes in 9- and 10- year old children: Utilizing the Adolescent Brain Cognitive Development ℠ (ABCD) Study
Source: PLoS One. 2021 Sep 8;16(9):e0256591. doi: 10.1371/journal.pone.0256591 (PMC8425530; doi:10.1371/journal.pone.0256591)
Supplement: S21 Table — Note. Starred regressions are significant at alpha .05. (DOCX) [file pone.0256591.s021.docx]

S21 Table. Internalizing symptoms regressed on various types of weekend screen time for Part 2, controlling for SES and race/ethnicity, separated by sex.

Standardized Partial

Beta t statistic p-value Std. Err. Correlation

Males (*N*=6071)

Parent Report 0.096 7.12 <.001* .059 .096

TV and Movies 0.052 3.85 <.001* .114 .052

Videos 0.057 4.19 <.001* .108 .056

Video Chat -0.006 -0.48 .632 .303 -.006

Texting 0.001 0.05 .964 .302 .001

Social Media 0.002 0.12 .905 .422 .002

Video Games 0.035 2.57 .010* .105 .035

Mature Video Games -0.005 -0.34 .734 .156 -.005

R-rated Movies -0.015 -1.12 .264 .225 -.015

Females (*N*=5598)

Parent Report 0.103 7.29 <.001* .064 .102

TV and Movies -0.009 -0.64 .526 .117 -.009

Videos 0.033 2.36 .018*  .119 .033

Video Chat -0.012 -0.84 .402 .279 -.012

Texting -0.024 -1.70 .089 .253 -.024

Social Media 0.024 1.73 .084 .304 .024

Video Games 0.030 2.12 .034* .141 .030

Mature Video Games 0.009 0.60 .550 .251 .008

R-rated Movies -0.008 -0.55 .583 .256 -.008

*Note*. Starred regressions are significant at alpha .05.
