# Supplementary material for: Screen time and early adolescent mental health, academic, and social outcomes in 9- and 10- year old children: Utilizing the Adolescent Brain Cognitive Development ℠ (ABCD) Study
Source: PLoS One. 2021 Sep 8;16(9):e0256591. doi: 10.1371/journal.pone.0256591 (PMC8425530; doi:10.1371/journal.pone.0256591)
Supplement: S22 Table — Note. Starred regressions are significant at alpha .05. (DOCX) [file pone.0256591.s022.docx]

S22 Table. Externalizing symptoms regressed on various types of weekend screen time for Part 2, controlling for SES and race/ethnicity, separated by sex.

Standardized Partial

Beta t statistic p-value Std. Err. Correlation

Males (*N*=6071)

Parent Report 0.083 6.18 <.001* .058 .083

TV and Movies 0.077 5.76 <.001* .112 .077

Videos 0.048 3.59 <.001* .107 .048

Video Chat 0.023 1.76 .079 .298 .024

Texting 0.001 0.05 .964 .217 .001

Social Media 0.053 3.99 <.001* .415 .054

Video Games 0.057 4.29 <.001* .057 .058

Mature Video Games 0.064 4.61 <.001* .153 .062

R-rated Movies 0.048 3.55 <.001* .221 .048

Females (*N*=5598)

Parent Report 0.107 7.62 <.001* .060 .106

TV and Movies 0.061 4.38 <.001* .109 .061

Videos 0.073 5.21 <.001* .110 .073

Video Chat 0.031 2.22 .027* .260 .031

Texting -0.024 -1.70 .089 .236 -.024

Social Media 0.078 5.63 <.001* .283 .078

Video Games 0.067 4.81 <.001* .132 .067

Mature Video Games 0.054 3.86 <.001* .234 .054

R-rated Movies 0.051 3.64 <.001* .238 .051

*Note*. Starred regressions are significant at alpha .05.
