# Supplementary material for: Screen time and early adolescent mental health, academic, and social outcomes in 9- and 10- year old children: Utilizing the Adolescent Brain Cognitive Development ℠ (ABCD) Study
Source: PLoS One. 2021 Sep 8;16(9):e0256591. doi: 10.1371/journal.pone.0256591 (PMC8425530; doi:10.1371/journal.pone.0256591)
Supplement: S23 Table — Note. Starred regressions are significant at alpha .05. (DOCX) [file pone.0256591.s023.docx]

S23 Table. Oppositional defiance disorder regressed on various types of weekend screen time for Part 2, controlling for SES and race/ethnicity, separated by sex.

Standardized Partial

Beta t statistic p-value Std. Err. Correlation

Males (*N*=6071)

Parent Report 0.081 6.03 <.001* .032 .081

TV and Movies 0.065 4.83 <.001* .061 .065

Videos 0.045 3.33 .001* .059 .045

Video Chat 0.024 1.78 .075 .164 .024

Texting 0.005 0.40 .689 .163 .005

Social Media 0.065 4.84 <.001* .228 .065

Video Games 0.054 3.98 <.001* .057 .053

Mature Video Games 0.048 3.47 .001* .084 .047

R-rated Movies 0.054 3.95 <.001* .121 .053

Females (*N*=5598)

Parent Report 0.082 5.83 <.001* .030 .082

TV and Movies 0.059 4.22 <.001* .055 .059

Videos 0.050 3.55 <.001* .055 .049

Video Chat 0.022 1.54 .124 .130 .021

Texting 0.031 2.24 .025* .117 .031

Social Media 0.077 5.48 <.001* .141 .076

Video Games 0.057 4.07 <.001* .066 .057

Mature Video Games 0.039 2.75 .006* .117 .038

R-rated Movies 0.043 3.01 .003* .119 .042

*Note*. Starred regressions are significant at alpha .05.
