# Supplementary material for: Screen time and early adolescent mental health, academic, and social outcomes in 9- and 10- year old children: Utilizing the Adolescent Brain Cognitive Development ℠ (ABCD) Study
Source: PLoS One. 2021 Sep 8;16(9):e0256591. doi: 10.1371/journal.pone.0256591 (PMC8425530; doi:10.1371/journal.pone.0256591)
Supplement: S24 Table — Note. Starred regressions are significant at alpha .05. (DOCX) [file pone.0256591.s024.docx]

S24 Table. Conduct disorder regressed on various types of weekend screen time for Part 2, controlling for SES and race/ethnicity, separated by sex.

Standardized Partial

Beta t statistic p-value Std. Err. Correlation

Males (*N*=6071)

Parent Report 0.079 5.93 <.001* .031 .080

TV and Movies 0.073 5.55 <.001* .059 .074

Videos 0.054 4.00 <.001* .056 .054

Video Chat 0.048 3.65 <.001* .157 .049

Texting 0.014 1.03 .305 .157 .014

Social Media 0.088 6.65 <.001* .218 .089

Video Games 0.056 4.19 <.001* .055 .056

Mature Video Games 0.073 5.29 <.001* .081 .071

R-rated Movies 0.067 4.99 <.001* .116 .067

Females (*N*=5598)

Parent Report 0.093 6.63 <.001* .032 .093

TV and Movies 0.071 5.16 <.001* .058 .072

Videos 0.072 5.11 <.001* .059 .071

Video Chat 0.046 3.30 .001* .138 .046

Texting 0.036 2.59 .010* .125 .036

Social Media 0.088 6.38 <.001* .150 .089

Video Games 0.074 5.38 <.001* .070 .075

Mature Video Games 0.082 5.85 <.001* .124 .081

R-rated Movies 0.084 6.01 <.001* .126 .084

*Note*. Starred regressions are significant at alpha .05.
