# Supplementary material for: Screen time and early adolescent mental health, academic, and social outcomes in 9- and 10- year old children: Utilizing the Adolescent Brain Cognitive Development ℠ (ABCD) Study
Source: PLoS One. 2021 Sep 8;16(9):e0256591. doi: 10.1371/journal.pone.0256591 (PMC8425530; doi:10.1371/journal.pone.0256591)
Supplement: S25 Table — Note. Starred regressions are significant at alpha .05. (DOCX) [file pone.0256591.s025.docx]

S25 Table. Attention problems regressed on various types of weekend screen time for Part 2, controlling for SES and race/ethnicity, separated by sex.

Standardized Partial

Beta t statistic p-value Std. Err. Correlation

Males (*N*=6071)

Parent Report 0.074 5.51 <.001*  .036 .074

TV and Movies 0.075 5.57 <.001* .068 .075

Videos 0.083 6.16 <.001* .065 .083

Video Chat 0.031 2.32 .020* .183 .031

Texting 0.012 0.91 .361 .182 .012

Social Media 0.039 2.88 .004* .254 .039

Video Games 0.076 5.66 <.001* .063 .076

Mature Video Games 0.026 1.88 .061 .094 .025

R-rated Movies 0.036 2.60 .009* .135 .035

Females (*N*=5598)

Parent Report 0.071 5.04 <.001* .035 .071

TV and Movies 0.054 3.87 <.001* .064 .054

Videos 0.096 6.79 <.001* .064 .094

Video Chat 0.027 1.93 .054 .152 .027

Texting 0.028 1.99 .046* .138 .028

Social Media 0.043 3.10 .002* .166 .043

Video Games 0.100 7.21 <.001* .077 .100

Mature Video Games 0.055 3.85 <.001* .137 .054

R-rated Movies 0.031 2.18 .029* .139 .030

*Note*. Starred regressions are significant at alpha .05.
