# Supplementary material for: Screen time and early adolescent mental health, academic, and social outcomes in 9- and 10- year old children: Utilizing the Adolescent Brain Cognitive Development ℠ (ABCD) Study
Source: PLoS One. 2021 Sep 8;16(9):e0256591. doi: 10.1371/journal.pone.0256591 (PMC8425530; doi:10.1371/journal.pone.0256591)
Supplement: S26 Table — Note. Starred regressions are significant at alpha .05. (DOCX) [file pone.0256591.s026.docx]

S26 Table. ADHD regressed on various types of weekend screen time for Part 2, controlling for SES and race/ethnicity, separated by sex.

Standardized Partial

Beta t statistic p-value Std. Err. Correlation

Males (*N*=6071)

Parent Report 0.094 6.98 <.001* .033 .094

TV and Movies 0.090 6.70 <.001* .063 .090

Videos 0.100 7.39 <.001* .060 .099

Video Chat 0.032 2.37 .018* .169 .032

Texting 0.010 0.72 .470 .169 .010

Social Media 0.048 3.55 <.001* .236 .048

Video Games 0.088 6.51 <.001* .059 .087

Mature Video Games 0.039 2.76 .006* .087 .037

R-rated Movies 0.040 2.91 .004* .126 .039

Females (*N*=5598)

Parent Report 0.076 5.41 <.001* .031 .076

TV and Movies 0.081 5.80 <.001* .057 .081

Videos 0.099 7.04 <.001* .058 .098

Video Chat 0.038 2.74 .006* .137 .038

Texting 0.050 3.60 <.001* .124 .050

Social Media 0.050 3.59 <.001* .149 .050

Video Games 0.101 7.27 <.001* .069 .101

Mature Video Games 0.072 5.07 <.001* .123 .071

R-rated Movies 0.039 2.74 .006* .125 .038

*Note*. Starred regressions are significant at alpha .05.
