# Supplementary material for: Screen time and early adolescent mental health, academic, and social outcomes in 9- and 10- year old children: Utilizing the Adolescent Brain Cognitive Development ℠ (ABCD) Study
Source: PLoS One. 2021 Sep 8;16(9):e0256591. doi: 10.1371/journal.pone.0256591 (PMC8425530; doi:10.1371/journal.pone.0256591)
Supplement: S27 Table — Note. Starred regressions are significant at alpha .05. (DOCX) [file pone.0256591.s027.docx]

S27 Table. Grades regressed on various types of weekend screen time for Part 2, controlling for SES and race/ethnicity, separated by sex.

Standardized Partial

Beta t statistic p-value Std. Err. Correlation

Males (*N*=6071)

Parent Report -0.088 -6.56 <.001* .005 -.092

TV and Movies -0.056 -4.13 <.001* .009 -.058

Videos -0.073 -5.36 <.001* .009 -.075

Video Chat -0.041 -3.05 .002* .023 -.043

Texting -0.036 -2.67 .008* .023 -.037

Social Media -0.039 -2.91 .004* .033 -.041

Video Games -0.057 -4.22 <.001* .008 -.059

Mature Video Games -0.136 -9.85 <.001* .012 -.137

R-rated Movies -0.099 -7.30 <.001* .017 -.102

Females (*N*=5598)

Parent Report -0.098 -6.95 <.001* .005 -.101

TV and Movies -0.047 -3.33 .001* .008 -.048

Videos -0.082 -5.80 <.001* .008 -.084

Video Chat -0.048 -3.45 .001* .020 -.050

Texting -0.042 -2.96 .003* .018 -.043

Social Media -0.056 -4.00 <.001* .021 -.058

Video Games -0.023 -1.64 .100 .010 -.024

Mature Video Games -0.078 -5.51 <.001* .017 -.080

R-rated Movies -0.067 -4.72 <.001* .018 -.068

*Note*. Starred regressions are significant at alpha .05.
