# Supplementary material for: Screen time and early adolescent mental health, academic, and social outcomes in 9- and 10- year old children: Utilizing the Adolescent Brain Cognitive Development ℠ (ABCD) Study
Source: PLoS One. 2021 Sep 8;16(9):e0256591. doi: 10.1371/journal.pone.0256591 (PMC8425530; doi:10.1371/journal.pone.0256591)
Supplement: S28 Table — Note. Starred regressions are significant at alpha .05. (DOCX) [file pone.0256591.s028.docx]

S28 Table. Average nightly hours of sleep regressed on various types of weekend screen time for Part 2, controlling for SES and race/ethnicity, separated by sex.

Standardized Partial

Beta t statistic p-value Std. Err. Correlation

Males (*N*=6071)

Parent Report -0.157 -12.29 <.001*  .004 -.164

TV and Movies -0.058 -4.47 <.001* .008 -.060

Videos -0.138 -10.69 <.001* .008 -.142

Video Chat -0.044 -3.39 .001* .022 -.046

Texting -0.059 -4.59 <.001* .022 -.062

Social Media -0.050 -3.90 <.001* .031 -.052

Video Games -0.103 -8.00 <.001* .008 -.107

Mature Video Games -0.128 -9.65 <.001* .011 -.129

R-rated Movies -0.095 -7.25 <.001* .016 -.097

Females (*N*=5598)

Parent Report -0.196 -14.86 <.001* .005 -.204

TV and Movies -0.072 -5.42 <.001* .008 -.075

Videos -0.143 -10.74 <.001* .008 -.148

Video Chat -0.038 -2.87 .004* .020 -.040

Texting -0.077 -5.79 <.001* .018 -.081

Social Media -0.070 -5.26 <.001* .022 -.073

Video Games -0.048 -3.64 <.001* .010 -.051

Mature Video Games -0.088 -6.51 <.001* .018 -.090

R-rated Movies -0.072 -5.33 <.001* .018 -.074

*Note*. Starred regressions are significant at alpha .05.
