# Supplementary material for: Screen time and early adolescent mental health, academic, and social outcomes in 9- and 10- year old children: Utilizing the Adolescent Brain Cognitive Development ℠ (ABCD) Study
Source: PLoS One. 2021 Sep 8;16(9):e0256591. doi: 10.1371/journal.pone.0256591 (PMC8425530; doi:10.1371/journal.pone.0256591)
Supplement: S29 Table — Note. Starred regressions are significant at alpha .05. (DOCX) [file pone.0256591.s029.docx]

S29 Table. Sleep disorder score regressed on various types of weekend screen time for Part 2, controlling for SES and race/ethnicity, separated by sex.

Standardized Partial

Beta t statistic p-value Std. Err. Correlation

Males (*N*=6071)

Parent Report 0.133 9.89 <.001* .045 .132

TV and Movies 0.059 4.36 <.001* .088 .059

Videos 0.084 6.17 <.001* .084 .083

Video Chat 0.022 1.64 .101 .234 .022

Texting 0.013 0.95 .340 .233 .013

Social Media 0.041 3.03 .002* .326 .041

Video Games 0.067 4.95 <.001* .081 .066

Mature Video Games 0.045 3.25 .001* .120 .044

R-rated Movies 0.030 2.23 .026* .174 .030

Females (*N*=5598)

Parent Report 0.143 10.23 <.001* .048 .142

TV and Movies 0.048 3.45 .001* .088 .048

Videos 0.065 4.62 <.001* .089 .064

Video Chat -0.003 -0.22 .829 .208 -.003

Texting 0.035 2.52 .012* .189 .035

Social Media 0.034 2.46 .014* .228 .034

Video Games 0.054 3.88 <.001* .106 .054

Mature Video Games 0.035 2.44 .015* .188 .034

R-rated Movies 0.028 1.95 .051 .191 .027

*Note*. Starred regressions are significant at alpha .05.
