# Supplementary material for: Screen time and early adolescent mental health, academic, and social outcomes in 9- and 10- year old children: Utilizing the Adolescent Brain Cognitive Development ℠ (ABCD) Study
Source: PLoS One. 2021 Sep 8;16(9):e0256591. doi: 10.1371/journal.pone.0256591 (PMC8425530; doi:10.1371/journal.pone.0256591)
Supplement: S30 Table — Note. Starred regressions are significant at alpha .05. (DOCX) [file pone.0256591.s030.docx]

S30 Table. Number of close friends who are boys regressed on various types of weekend screen time for Part 2, controlling for SES and race/ethnicity, separated by sex.

Standardized Partial

Beta t statistic p-value Std. Err. Correlation

Males (*N*=6071)

Parent Report -0.018 -1.33 .184 .037 -.018

TV and Movies 0.025 1.82 .070 .070 .024

Videos 0.049 3.56 <.001* .067 .048

Video Chat 0.071 5.28 <.001* .186 .071

Texting 0.085 6.32 <.001* .185 .085

Social Media 0.073 5.35 <.001* .259 .072

Video Games 0.069 5.06 <.001* .065 .068

Mature Video Games 0.083 5.89 <.001* .095 .079

R-rated Movies 0.032 2.36 .019* .138 .032

Females (*N*=5598)

Parent Report 0.028 1.97 .049* .015 .028

TV and Movies 0.032 2.26 .024* .028 .032

Videos 0.054 3.84 <.001* .028 .053

Video Chat 0.049 3.52 <.001* .066 .049

Texting 0.053 3.79 <.001* .060 .053

Social Media 0.058 4.16 <.001* .072 .058

Video Games 0.063 4.48 <.001* .034 .062

Mature Video Games 0.101 7.12 <.001* .059 .099

R-rated Movies 0.065 4.59 <.001* .061 .064

*Note*. Starred regressions are significant at alpha .05.
