# Supplementary material for: Screen time and early adolescent mental health, academic, and social outcomes in 9- and 10- year old children: Utilizing the Adolescent Brain Cognitive Development ℠ (ABCD) Study
Source: PLoS One. 2021 Sep 8;16(9):e0256591. doi: 10.1371/journal.pone.0256591 (PMC8425530; doi:10.1371/journal.pone.0256591)
Supplement: S31 Table — Note. Starred regressions are significant at alpha .05. (DOCX) [file pone.0256591.s031.docx]

S31 Table. Number of close friends who are girls regressed on various types of weekend screen time for Part 2, controlling for SES and race/ethnicity, separated by sex.

Standardized Partial

Beta t statistic p-value Std. Err. Correlation

Males (*N*=6071)

Parent Report -0.016 -1.17 .241 .025 -.016

TV and Movies 0.011 0.80 .422 .047 .011

Videos 0.035 2.56 .010* .045 .035

Video Chat 0.056 4.12 <.001* .126 .056

Texting 0.066 4.92 <.001* .125 .066

Social Media 0.109 8.06 <.001* .174 .108

Video Games 0.032 2.34 .019* .044 .031

Mature Video Games 0.052 3.71 <.001* .065 .050

R-rated Movies 0.042 3.10 .002* .093 .042

Females (*N*=5598)

Parent Report 0.038 2.65 .008* .046 .037

TV and Movies 0.021 1.50 .135 .084 .021

Videos 0.029 2.06 .039* .085 .029

Video Chat 0.056 4.13 <.001* .198 .056

Texting 0.051 3.64 <.001* .180 .051

Social Media 0.041 2.90 .004* .217 .040

Video Games 0.038 2.69 .007* .101 .037

Mature Video Games 0.033 2.32 .020* .179 .032

R-rated Movies 0.028 1.94 .052 .183 .027

*Note*. Starred regressions are significant at alpha .05.
