# Supplementary material for: Screen time and early adolescent mental health, academic, and social outcomes in 9- and 10- year old children: Utilizing the Adolescent Brain Cognitive Development ℠ (ABCD) Study
Source: PLoS One. 2021 Sep 8;16(9):e0256591. doi: 10.1371/journal.pone.0256591 (PMC8425530; doi:10.1371/journal.pone.0256591)
Supplement: Attachment — Submitted filename: Comments_PONE-D-20-40267.pdf [file pone.0256591.s032.pdf]

Comments to Manuscript Number PONE-D-20-40267

Full Title: Screen time is only modestly associated with mental health, academic outcomes, and peer relationships in the Adolescent Brain Cognitive Development ℠ Study

Short Title: Screen time and the ABCD Study

The paper examines an interesting and useful topic related to screen time and mental health, behavioral problems, academic performance, sleep habits, and peer relationships in the USA. It does fit the scope of *PLoS ONE*. Overall, it contributes to the advancement of knowledge and debate on matters of mental anxiety, depression, and academic results by those who are using more screens in the United States. Though the study finding got a small effect size. But still, the results are significant in line with the expected hypothesis. The findings will help to understand to what extent screen time are creating vulnerability to the child during their early adolescent stage.

However, the fundamental shortcomings of the paper are:

1. Why the study is divided by study 1 and study 2 in the results section. I understand study 1 is for weekday screen time and study 2 for weekend screen time. Changing it to part 1 and part 2 would help. Need a clear justification for dividing these two parts.
2. For logistics regression, the study divides the sample by their sex. The study runs two separate regressions for both males and females. Some further explanation and justification need to be provided regarding the two separate regressions. It would be interesting if the author(s) could add a combined regression (for both male and female) using sex as a dummy explanatory variable and then run two separate regression for male and female to check how does gender play a role in explaining the effect of screen time on mental health, academic performance and peer relationships. The study is restricting the sample for two separate regression by their sex and in fact, sex is a channel to explain the role of screen time on different outcome variables.
3. The study found a very low effect size to explain the linkage between total screen time and the outcome measures. Along with the total screen time, the study can estimate the effect of screen time by creating a dummy for an acceptable level of screen time and beyond that. This will help to show how too much screen time creating an effect on the outcome variables.
4. The descriptive statistics about the outcome and explanatory variables should be provided in the main paper instead of placing them in the appendix. Suggest bringing table S1 and S2 after combining them in a single table up into the main body of the text so the reader has a better sense of their characteristics (if the appendix is published with the paper (and not just online) that may be less of a problem).
5. It would be better to provide the overall descriptive statistics about the SES.
6. Reference to other studies, including Twenge and Campbell, 2018; Oswald et al., 2020.

7. Better to use the clustering by different demographic zone while running a regression. As the study participant covers different demographic zone, therefore, the clustering (clustered standard errors) could provide more robust results.

8. The discussion section is written appropriately. However, the results section is not written consistently. It would be better to make the writing of the result section consistent to make it more reader-friendly.

9. Though the study focused too much on sex and weekday/weekend without proper justification. A clear justification is useful to add.

10. The study considers many outcomes without focusing on them in more detail. It would be better if the study restricts their outcome variable and then cover the heterogeneous channels to find the linkage between the outcome and screen time. For example, sex is a channel where the effect size of screen time on the outcome variables is different depending on the sex of the participants. The study could concentrate on some other channels from the SES to find the effect of screen time on the outcome variables in more detail.
