# Supplementary material for: Screen time and early adolescent mental health, academic, and social outcomes in 9- and 10- year old children: Utilizing the Adolescent Brain Cognitive Development ℠ (ABCD) Study
Source: PLoS One. 2021 Sep 8;16(9):e0256591. doi: 10.1371/journal.pone.0256591 (PMC8425530; doi:10.1371/journal.pone.0256591)
Supplement: Attachment — Submitted filename: Re_Comments_PONE-D-20-40267.pdf [file pone.0256591.s035.pdf]

Comments to Manuscript Number PONE-D-20-40267

Full Title: Screen time and early adolescent mental health, academic, and social outcomes in 9- and 10- year old children: Utilizing the Adolescent Brain Cognitive Development ℠

(ABCD) Study

Short Title: Screen time and the ABCD Study

Thanks to the authors for addressing most of the comments that were made during the first review. Still, I have few comments to fit the paper as a good one to publish in *PLoS ONE*. Therefore, the paper should address the existing shortcomings:

1. How is the variable sex dummy constructed? Which one is the base category to compare with?
2. The authors have used the interaction of sex and screen time (weekday/weekend) and mostly got insignificant results. But they somehow missed the main interpretation of interaction terms. Why the coefficient of interaction is insignificant while they are highly significant separately? In the discussion section, the authors have written few sentences on this issue. However, it requires more discussion on it as the existing write-up may create confusion.
3. The paper mostly focused on sex as an explanatory variable along with screen time. But this lacks concentration on SES and race/ethnicity in results and discussion. Adding **race/ethnicity and SES** by **creating dummies** will increase the scope and contribution of this paper. As they are included in each model but not reported. Therefore, reporting them in main results, particularly, in table 2 and table 4 (of the revised submission) similar to sex would be much appreciated.
4. One of the concerns was multicollinearity. The author says there is no multicollinearity referring to the appendix table 3 where the correlation among the variables are reported including the sets of outcome and explanatory variables **without reporting SES and race/ethnicity** (these are included in regression table). Better to produce tables with multicollinearity tests for models used in this paper (alternative to correlation table).
