# Supplementary material for: Screen time and early adolescent mental health, academic, and social outcomes in 9- and 10- year old children: Utilizing the Adolescent Brain Cognitive Development ℠ (ABCD) Study
Source: PLoS One. 2021 Sep 8;16(9):e0256591. doi: 10.1371/journal.pone.0256591 (PMC8425530; doi:10.1371/journal.pone.0256591)
Supplement: Attachment — Submitted filename: Comments_third time_PONE-D-20-40267.pdf [file pone.0256591.s037.pdf]

Comments to Manuscript Number PONE-D-20-40267R2

Full Title: Screen time and early adolescent mental health, academic, and social outcomes in 9- and 10- year old children: Utilizing the Adolescent Brain Cognitive Development ℠ (ABCD) Study

Short Title: Screen time and the ABCD Study

Thanks to the authors for their effort to address all the comments and implement the recommendations that were made during the second time review. I appreciate their work to make the manuscript reads better. I think this version now fits as a good one to publish in *PLoS ONE*. I believe the changes they have made significantly improved the quality of this paper.
