# Supplementary material for: Screen time and early adolescent mental health, academic, and social outcomes in 9- and 10- year old children: Utilizing the Adolescent Brain Cognitive Development ℠ (ABCD) Study
Source: PLoS One. 2021 Sep 8;16(9):e0256591. doi: 10.1371/journal.pone.0256591 (PMC8425530; doi:10.1371/journal.pone.0256591)
Supplement: Attachment — Submitted filename: Review Comments.docx [file pone.0256591.s033.docx]

I enjoyed reading this article and drawing conclusion from larger sample size is commendable. Also the study pointed out the influence of screen time on academic outcomes and others which is very insightful. However, I have few comments and suggestions for them.

The title “Screen time is only modestly associated with mental health, academic outcomes, and peer relationships in the Adolescent Brain Cognitive Development” should be reshaped. A good title should at least, tell us the dependent and independent variables, study population and the area of study. The title looks a bit confusing.

Abstract section

“We are using screens more than ever” [line 23]. This is not clear. Please the “we” should be clarified. Who are you referring to?

Main Text

Introduction

“with 95% of teens having access to a smartphone”…. [line 45]. The “95%”, is it a global prevalence or what?

The authors did well by stating the expected results/working hypothesis. However, the study lacks theoretical conceptual framework. Therefore, I suggest the authors should add a theory to it.

Also, authors should tell us the prevalence of screen time for us to be clear about proportion of children being exposed to screen, from global to study area perspective, if such data exist. Such trend analysis could enrich the paper.

Statistical analysis

Why should the authors use Multiple linear regressions because such estimating technique may not help to understand differences within groupings? Also, they fail to account or check for multicollinearity that might exist between explanatory variables.

Also, they should simply tell us how the results were interpreted.

Discussion section

The authors did a great job by comparing their results with previous studies. However, their explanations were mostly based on conjecture and speculations without literature. I suggest the authors adopt/adapt a theory and situate their results and discussions in the theory.

Also, at the introductory aspect of the discussion, I suggest the authors should tell us the main/key findings in brief and show us how significant are these results before moving on to discuss them.

References

The authors also used current literature which is commendable.

Overall, the paper could be published if they are able to improve the paper.

Also, they should proof read for few grammatical errors.
