# Supplementary material for: Screen time and early adolescent mental health, academic, and social outcomes in 9- and 10- year old children: Utilizing the Adolescent Brain Cognitive Development ℠ (ABCD) Study
Source: PLoS One. 2021 Sep 8;16(9):e0256591. doi: 10.1371/journal.pone.0256591 (PMC8425530; doi:10.1371/journal.pone.0256591)
Supplement: Attachment — Submitted filename: Response to Reviewers.docx [file pone.0256591.s036.docx]

Response to Reviewers

**We appreciate the opportunity to revise and resubmit our manuscript; incorporating the reviewer suggestions below has improved the quality of our paper, and we thank you for the additional consideration.**

***Please note that all references to line number are within the Revised Manuscript with Track Changes.**

Reviewer #1: Comments to Manuscript Number PONE-D-20-40267

Full Title: Screen time and early adolescent mental health, academic, and social outcomes in 9- and 10- year old children: Utilizing the Adolescent Brain Cognitive Development ℠

(ABCD) Study

Short Title: Screen time and the ABCD Study

Thanks to the authors for addressing most of the comments that were made during the first review. Still, I have few comments to fit the paper as a good one to publish in PLoS ONE. Therefore, the paper should address the existing shortcomings:

1. How is the variable sex dummy constructed? Which one is the base category to compare with?

**The following has been added to the Statistical Analysis section of the Materials and Methods at line 306: Sex was dummy coded with females = 0 and males = 1, making “females” the base category for comparison.**

2. The authors have used the interaction of sex and screen time (weekday/weekend) and mostly got insignificant results. But they somehow missed the main interpretation of interaction terms. Why the coefficient of interaction is insignificant while they are highly significant separately? In the discussion section, the authors have written few sentences on this issue. However, it requires more discussion on it as the existing write-up may create confusion.

**Thank you for urging clarification on these interpretations.**

**The beginning of the Discussion section at line 461 now reads:** **These results have important implications. The lack of consistently significant interactions between screen time and sex—but often significant main effects for both screen time and sex—demonstrate that generally, both screen time and sex predict the outcome variables, but that the effect of screen time on the outcome variables often does not depend on sex, and vice versa. For the outcome measures with non-significant interaction terms but significant main effects of both/either screen time and/or sex, it appears that screen time and sex are independent predictors of the outcome measure. For these outcome measures, the effect of either screen time or sex on the outcome variable did not depend on the other independent variable. A potential reason for that finding could be sex differences in how screens are being used. The only outcome measure demonstrating a significant interaction term, for Part 1 and for Part 2, is number of close friends who are males. It is possible that, because males in this study tend to use screen time for video gaming—which is often a social activity—more than females do (refer to Table 1), screen time and sex interact such that the effect of screen time (e.g., using screens for video gaming) on number of close male friends depends on the sex of the participant, where male participants who spend more time on screens video gaming have more male friends.**

3. The paper mostly focused on sex as an explanatory variable along with screen time. But this lacks concentration on SES and race/ethnicity in results and discussion. Adding race/ethnicity and SES by creating dummies will increase the scope and contribution of this paper. As they are included in each model but not reported. Therefore, reporting them in main results, particularly, in table 2 and table 4 (of the revised submission) similar to sex would be much appreciated.

**Thank you for this suggestion. Both race/ethnicity and SES are already dummy coded, as they are categorical variables rather than continuous variables.**

**For SES, the coding scheme is as follows: 1 = < $5,000; 2 = $5,000 - $11,999; 3 = $12,000 - $15,999; 4 = $16,000 - $24,999; 5 = $25,000 - $34,999; 6 = $35,000 - $49,999; 7 = $50,000 - $74,999; 8 = $75,000 - $99,999; 9 = $100,000 - $199,999; and 10 = $200,000+; with options for “don’t know” and “refuse to answer.” This information has been added to line 291 of the Combined family income explanation of the Measures section of the Materials and Methods.**

**For race/ethnicity, the coding scheme is as follows:** **1 = White; 2 = Black; 3 = Hispanic; 4 = Asian; 5 = Other. This information has been added to line 297 of the Race/Ethnicity explanation of the Measures section of the Materials and Methods.**

**We have followed the suggestion to report statistics for both race/ethnicity and SES, as we agree that including them will increase the scope and contribution of this paper. Please refer to Table 2 and Table 4 (of the revised/latest submission) to find the reported results.**

**The following has also been added to lines 349 and 408 just prior to Table 2 and Table 4:** **Our primary interest was examination of the effects of screen time and sex on our dependent variables; however, we also report results for race/ethnicity and SES for the sake of completeness. The main effect of SES was also often significant.**

**We discuss the significance of the main effect of SES in the Discussion section at line 480: However, the effect of screen time was small (<2% of the variance explained) for all outcomes, with SES—which was demonstrated to be a significant predictor for the nearly all outcome variables of interest— accounting for much more of the variance (~5%), perhaps because parent SES contributes to nearly every facet of children’s physical and mental health outcomes [28].**

4. One of the concerns was multicollinearity. The author says there is no multicollinearity referring to the appendix table 3 where the correlation among the variables are reported including the sets of outcome and explanatory variables without reporting SES and race/ethnicity (these are included in regression table). Better to produce tables with multicollinearity tests for models used in this paper (alternative to correlation table).

**Thank you for this suggestion. We have conducted multicollinearity tests via examination of tolerance and variance inflation factor (VIF), and have produced two new tables displaying those results for the Supporting Information, S3 Table and S5 Table. We chose to retain the correlation tables (S2 Table and S4 Table) in order to provide readers with both the multicollinearity test results as well as the correlation matrices, in case both tables are of interest to readers. The results of both the multicollinearity tests and the correlation tables show that our variables do not demonstrate multicollinearity.**

**The following has also been added to the Part 1 section of the Results at line 345: The data do not demonstrate multicollinearity, as seen in S3 Table.**

**The following has been added to the Part 2 section of the Results at line 403: The data do not demonstrate multicollinearity, as seen in S5 Table.**

**Please note that the original S3 table (showing correlations between variables for Part 2) has been renamed to S4 Table, and subsequent Supporting Information Tables have been renamed accordingly and referred to accordingly in the manuscript. The changes allow the Supporting Information Tables to be referred to in numeric order.**
